# Electromigration Mechanism of Failure in Flip-Chip Solder Joints Based on Discrete Void Formation

**DOI:** 10.1038/s41598-017-06250-8

**Published:** 2017-12-20

**Authors:** Yuan-Wei Chang, Yin Cheng, Lukas Helfen, Feng Xu, Tian Tian, Mario Scheel, Marco Di Michiel, Chih Chen, King-Ning Tu, Tilo Baumbach

**Affiliations:** 1 Institute for Photon Science and Synchrotron Radiation (IPS), Karlsruhe Institute of Technology (KIT), Eggenstein-Leopoldshafen, 76344 Germany; 2 Department of Materials Science and Engineering, National Chiao Tung University, Hsin-chu, 30010 Taiwan, R.O.C.; 3 Laboratory for Applications of Synchrotron Radiation (LAS), Karlsruhe Institute of Technology (KIT), Karlsruhe, 76049 Germany; 4 ESRF - The European Synchrotron, 71 avenue des Martyrs, 38000 Grenoble, France; 5 Department of Materials Science and Engineering, UCLA, Los Angeles, California, 90095-595 USA; 6 Synchrotron SOLEIL, L’Orme des Merisiers Saint-Aubin, 91192 Gif-sur-Yvette, France

## Abstract

In this investigation, SnAgCu and SN100C solders were electromigration (EM) tested, and the 3D laminography imaging technique was employed for *in-situ* observation of the microstructure evolution during testing. We found that discrete voids nucleate, grow and coalesce along the intermetallic compound/solder interface during EM testing. A systematic analysis yields quantitative information on the number, volume, and growth rate of voids, and the EM parameter of DZ*. We observe that fast intrinsic diffusion in SnAgCu solder causes void growth and coalescence, while in the SN100C solder this coalescence was not significant. To deduce the current density distribution, finite-element models were constructed on the basis of the laminography images. The discrete voids do not change the global current density distribution, but they induce the local current crowding around the voids: this local current crowding enhances the lateral void growth and coalescence. The correlation between the current density and the probability of void formation indicates that a threshold current density exists for the activation of void formation. There is a significant increase in the probability of void formation when the current density exceeds half of the maximum value.

## Introduction

In the last decade flip-chip (FC) technology has become one of the most important packaging technologies, providing increased packaging density, and as a result, increased miniaturisation, reduced power consumption and faster electronic devices. Many studies have been carried out on the lead (Pb)-free solder due to the rising environmental concerns^1–6^. Of the various kinds of Pb-free solders, Sn3.0Ag0.5Cu (SAC305) is most popular because of its great performance in mechanical tests such as thermal cycling^7–9^. Because the precipitation of Ag_3_Sn on the grain boundaries pins down the growth of grains, both the strength and the ductility of the solder joint are enhanced. However, the high cost of Ag encourages the development of alternative alloys to replace the SAC alloy. One approach is to lower the Ag composition below the single digit, which has led to alloys such as Sn2.0Ag0.5Cu (SAC205) and Sn1.0Ag0.5Cu (SAC105)^8^. Another approach is based on the Sn-Cu system with further additives or dopants. The addition of Ni, Mn, and Ti is used to achieve better performance in both the drop and thermal cycling tests^10^. Moreover, the addition of Ge (SN100C) is now widely applied to Pb-free wave soldering^7^. Apart from the mechanical reliability performance however, other reliability issues such as electromigration (EM) are not well studied. To date, only a few studies have focused on the EM reliability of SN100C solder^11^.

The small size of FC solder bump worsens the EM reliability issues due to current crowding^5,12^. Moreover, the line-to-bump geometry, the composite materials, and the anisotropic material properties of Sn make the analysis of EM failure mechanisms very difficult. In initial studies there were two major failure mechanisms reported: the pancake void propagation^13–15^ and the dissolution of under-bump-metallization (UBM)^16–18^. More recently a third failure mode the discrete void formation and growth in one type of solder has been identified using synchrotron laminography, a non-destructive 3D volume imaging technique^19^. The pancake void propagation model describes the depletion of Sn and the consequent void growth along the interface between solder and the intermetallic compounds (IMC) which are below the UBM. When the dissolution of UBM is faster than the depletion of Sn, open-circuit failure occurs between the UBM and the top trace. Instead of the single void in pancake void model, the discrete voids model describes the simultaneous nucleation and growth of multiple voids at the IMC/solder interface. However, the conventional way to observe the microstructure evolution *in-situ* during EM testing is to polish the sample to the desired cross section before testing^20–23^. This intervention inevitably alters the boundary conditions during testing since the polished interface becomes a free surface, where surface oxidation, change of heat dissipation, and the relief of stresses can all be introduced. Moreover, the diffusion at the exposed surface is much faster than the lattice diffusion, which will significantly change the failure mechanism^24^. To avoid such artificial effects, three-dimensional (3D) synchrotron X-ray tomography^25,26^ or laminography^27,28^ techniques have been employed for non-destructive observations, but few of these have achieved quantitative *in-situ* analysis on microelectronic devices under conditions of practical operation^11,19^. To inspect entire electronic devices^29^ without extracting and preparing samples laminography has certain advantages over tomography^30^, and it has already proven its usefulness in different scientific fields such as biology^31^, paleontology^32^, material science^33,34^ or cultural heritage studies^35^.

In our study, five samples with two types of UBM and two compositions of solder were EM tested with two different magnitudes of current density. The two UBMs were Ti/Cu/Cu and electroless Ni/Au, which are abbreviated as Cu and Ni in this study while the two solder types, SN100C and SAC1205, are abbreviated as SN and SAC, respectively. In summary, the five samples are marked as Cu-SN-L, Cu-SAC-L, Cu-SAC-H, Ni-SN-L, and Ni-SN-H (see also Table 1), where L and H represent the low and high currents applied. In order to visualize the solder interior and the interface to the UBM without intervention or special sample preparation, hence preserving the boundary conditions of testing, we employed 3D laminography for *in-situ* observation of the EM failure process during EM testing. Following all the stages of EM process, we have verified that the third EM failure mechanism (i.e. discrete void formation) occurs in all samples. This is confirmed based on the quantitative analysis of void growth, obtained from the laminography reconstructed volumes at different stages. Although the growth of void in all samples follows the Johnson-Mehl-Avarmi (JMA) theory of phase transformation, we do not find a good agreement with respect to their number, growth rate, and spatial distribution. Finally, several series of finite-element (FE) model were built based on the 3D laminography volumes. With the FE analysis the interactions between the microstructure evolution and the current density distribution were investigated.

**Table 1:** The DZ* of all samples obtained from mass transportation and the slope of the lines in Fig. 2(c).

Sample	UBM	Solder	DZ* (cm^2^/s)	Slope	Error of Slope
Cu-SN-L	Ti/Cu/Cu	SN100C	6.87 × 10^−11^	0.74	0.03272
Cu-SAC-L	Ti/Cu/Cu	SAC1205	1.40 × 10^−10^	0.58	0.03215
Cu-SAC-H	Ti/Cu/Cu	SAC1205	1.15 × 10^−10^	0.48	0.02676
Ni-SN-L	E-less Ni/Au	SN100C	4.15 × 10^−11^	0.78	0.05844
Ni-SN-H	E-less Ni/Au	SN100C	8.36 × 10^−11^	0.70	0.01541

## Results

### Electromigration failure due to growth and coalescence of discrete voids

**Figure 1:**
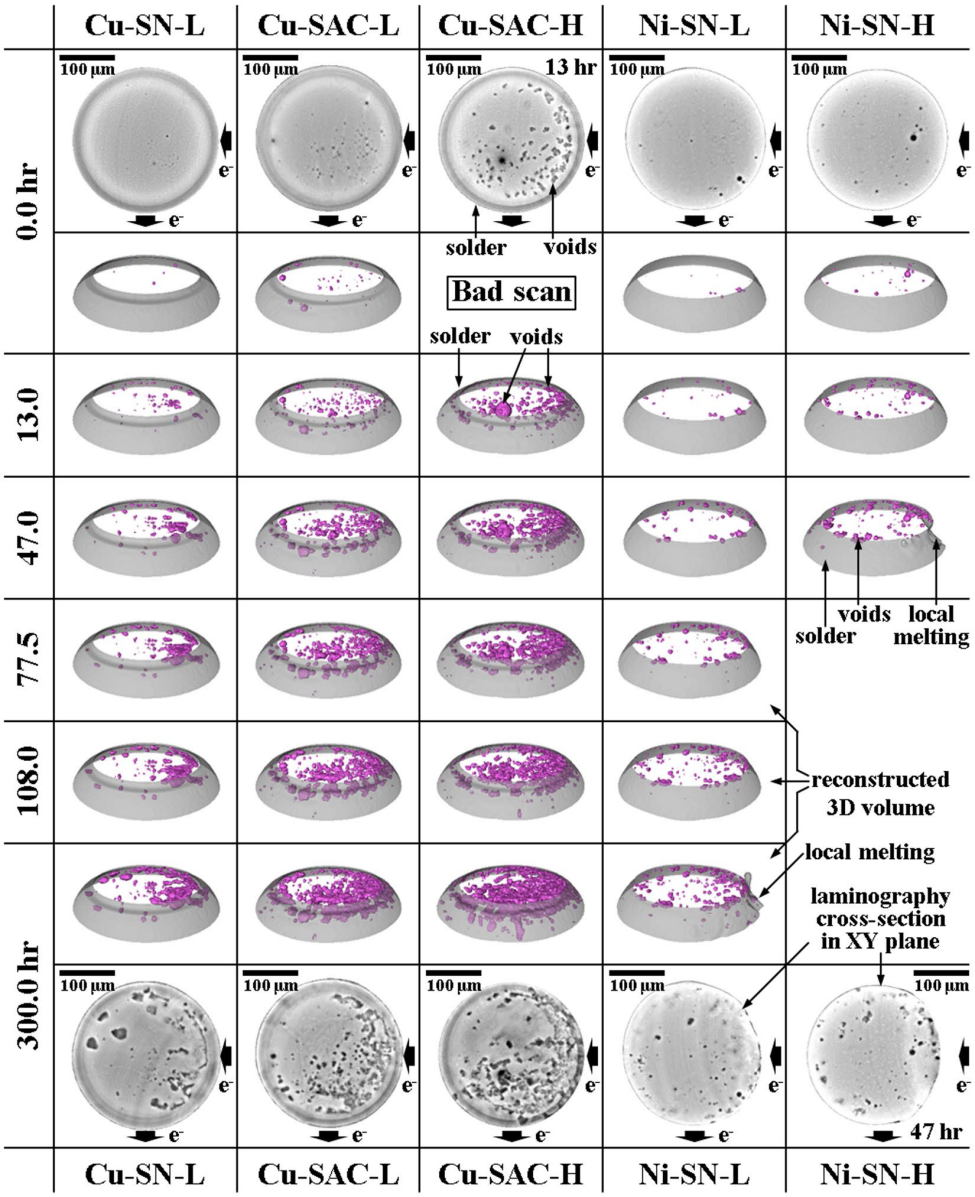
The reconstructed cross-sectional laminography images at the UBM/solder interface before (initial state) and after EM testing for 300 hours (on the top and the bottom, respectively). The solder material is visible in gray and the voids in black. In the middle: 3D rendition of the top portion of the solder joints, showing the evolution of discrete voids as a function of EM testing time; the solder outline is rendered in semi-transparent gray while the voids are in magenta.

In all the tested material combinations, in contrast to the well-known pancake void model^13–15^, we observed the new third failure mechanism of discrete void growth and coalescence^19^. We observe that discrete voids continue nucleating and growing along the IMC/solder interface throughout the testing period, as shown in the initial and late stages of plane-view cross-sectional X-ray laminography images in Fig. 1, on the top and on the bottom respectively.

The evolution of the discrete voids is clearly visualized by comparison of the laminography images at 0 hr and 300 hr. Furthermore, in Cu-SAC-L and Cu-SAC-H some voids coalesce into several large ones on the right-hand side of the bump. The coalescence results from the current crowding effect and the intrinsic faster diffusion of the SAC1205 solder (compared to SN100C), as will be further discussed in the following sections. 3D volume rendering is aligned with these laminography cross sections, as shown in the middle part of Fig. 1, for the demonstration of the discrete void growth and coalescence process at different EM testing stages.

In addition we also note a significant shape change in Ni-SN-H due to local melting at 47 hr, leading to the failure of this solder. For other type of solders, after some 100 hr of testing, the discrete voids grow and coalesce into large voids, leading to the failure of the solder joint. Quantitative analysis based on the *in-situ* 3D laminography measurements are presented and discussed in the following sections with more details.

### The volume of voids

**Figure 2:**
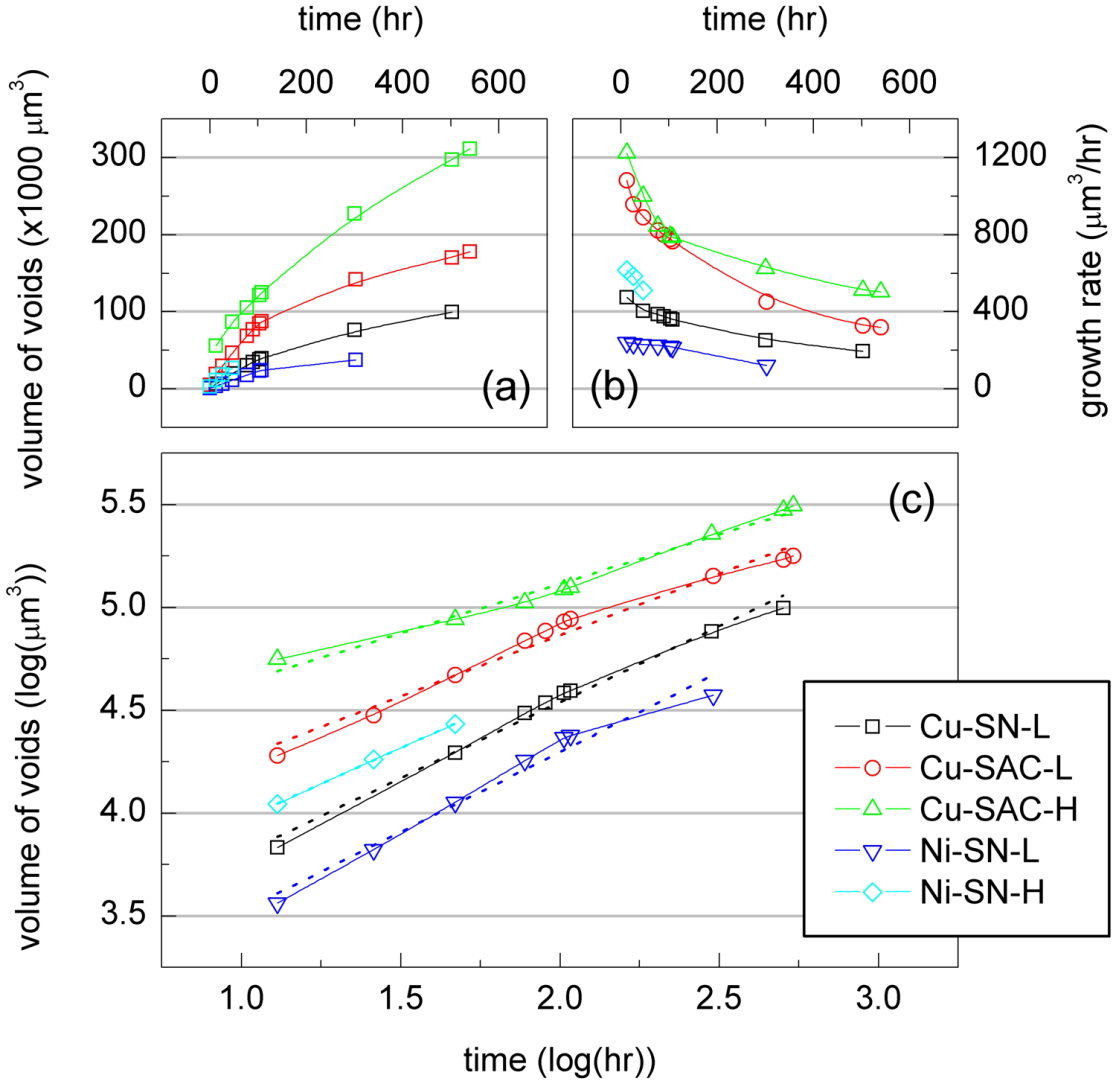
(**a**) The evolution of total volume of voids in each sample during EM testing; (**b**) the growth rate of total void volume in each sample; (**c**) logarithmic plot of (*a*).

Figure 2(a) shows the evolution of the total volume of voids as a function of the EM testing time for all samples. All the curves follow a power-law evolution where the volume increase of voids during testing can be expressed by

1 $${\rm{\Delta }}V={V}_{t}-{V}_{0}=R{t}^{n}$$

where *V*_0_ is the initial volume of voids, *V*_*t*_ is the total volume of voids at testing time *t*, *R* is a growth constant, and *n* is the exponent of growth. Taking the logarithm yields

2 $$\mathrm{log}({\rm{\Delta }}V)=\,\mathrm{log}(R)+n\,\mathrm{log}(t)$$

i.e. when plotting the porosity change in a double-logarithmic manner, we can directly determine the growth exponent *n* from the slope of the fitting line, together with *R* from the intercept. The exponent of growth represents the dominant mechanism of void growth. In order to analyze the kinetics Fig. 2(a) is redrawn with a logarithmic axis, to obtain Fig. 2(c). The intercept and slope are listed in Table 1. According to previous studies, *n* equal to 1.0 implies that the reaction is dominated by interfacial reaction^36,37^, while if *n* equal to 0.5, the reaction is controlled by the diffusion of species. As shown in Table 1, for Cu-SAC-L and Cu-SAC-H *n* is close to 0.5, which means that Sn diffusion dominates the void formation in SAC1205 solder^38–40^. In contrast, in the case of Cu-SN-L, Ni-SN-L, and Ni-SN-H samples, in which the SN100C solder is used, *n* is found to lie between 0.70 and 0.78, implying that besides Sn diffusion, the nucleation of voids may play an important role during EM tests.

The product of diffusivity and effective charge number, DZ*, is one of the most important material properties in EM. In solder, the diffusion flux of Sn driven by EM can be represented by^41^

3 $${J}_{{\rm{EM}}}=C\frac{D}{kT}{Z}^{\ast }e\rho j$$

where *J*_EM_ is the flux of EM, *C* is the concentration of Sn, *k* is the Boltzmann constant, *T* is the absolute temperature, *e* is the electric charge, *ρ* is the resistivity, and *j* is the current density. When the EM results in void formation, we have

4 $${J}_{{\rm{EM}}}=\frac{{V}_{{\rm{EM}}}}{{\rm{\Omega }}At}$$

where *V*_EM_ is the volume of voids, Ω is the atomic volume of Sn, *A* is the contact area, and *t* is the testing time. Combining Eqs (3) and (4), and knowing that Ω equals to 1/*C*, DZ* can be obtained as

5 $$D{Z}^{\ast }=\frac{k}{Ae\rho }\frac{{V}_{{\rm{EM}}}T}{tj}$$

The DZ* values calculated according to Eq. (5) are listed in Table 1 for each material combination. The calculation of DZ* supports the above conclusion. It is seen that the DZ* value of SAC1205 is about twice that of SN100C, implying that the voids grow much faster in SAC1205 than in SN100C. These observations may also be attributed to the smaller grain size in SAC1205^42^.

### The projected void area

**Figure 3:**
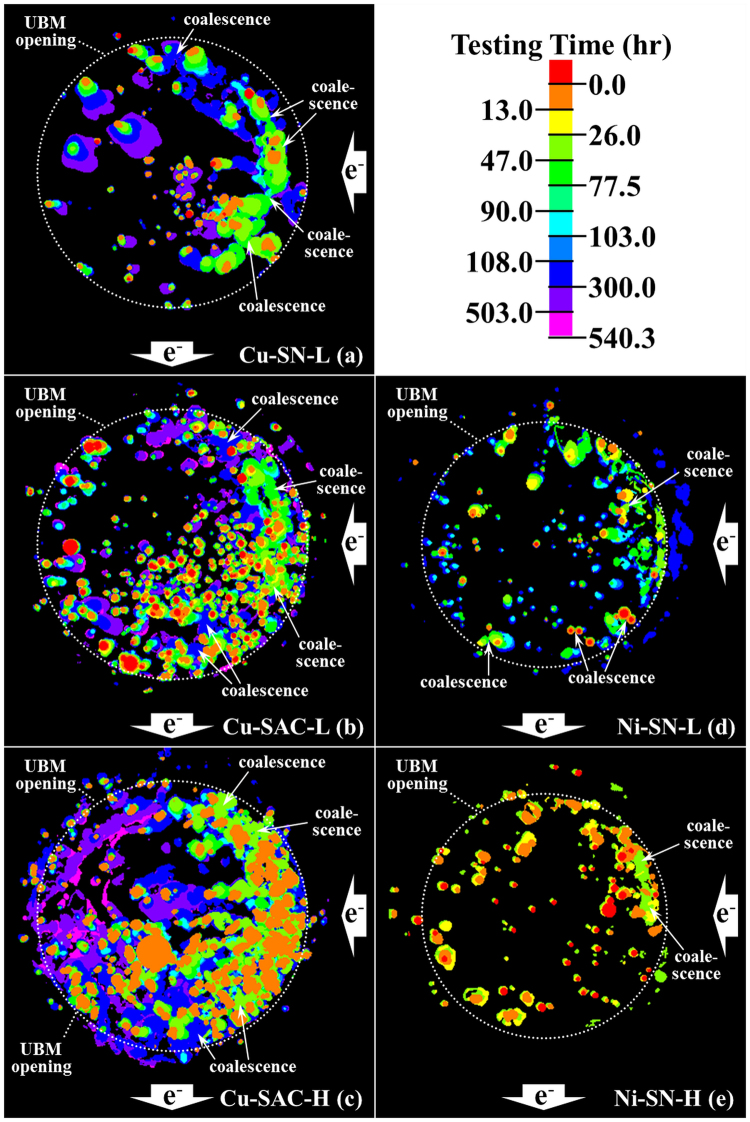
The superimposed images of the projection area of all voids onto the UBM opening of different EM testing stages: (**a**) Cu-SN-L, (**b**) Cu-SAC-L, (**c**) Cu-SAC-H, (**d**) Ni-SN-L, and (**e**) Ni-SN-H. The voids formed at a given testing stage are coded in specific colors as indicated by the colorbar.

Since most of the voids grow along the IMC/solder interface, in order to illustrate the microstructure evolution more clearly, we project the 3D void shapes onto the UBM opening plane for each EM stage and then overlap all the stages by using different colors, as shown in Fig. 3. Unlike Cu-SN-L, Cu-SAC-L, and Cu-SAC-H, the coalescence between voids is not significant in Ni-SN-L and Ni-SN-H. This indicates that the electroless Ni/Au UBM in Ni-SN-L and -H samples suppresses or retards void growth. The increase of the total void volume in Ni-SN-L and -H mainly results from the formation of new voids. In contrast, the coalescence between voids in Cu-SN-L, Cu-SAC-L and -H is very pronounced. Besides the coalescence, void nucleation is also severe in both Cu-SAC-L and -H.

From the viewpoint of spatial void distribution, more voids have nucleated in the right-hand side of the solder joint, which is the entrance point of the electron flow and correspondingly the point of current crowding^13^. In addition, voids mainly start forming in the periphery and then grow towards the center of the UBM because both the thick Cu and Ni UBM cause the secondary current crowding^43^ effect: The resistivity of Cu and Ni are 1.7 and 6.8 μΩ⋅cm, respectively, which are much smaller than that of Sn at 14.6 μΩ⋅cm. Therefore, the thicker Cu/Ni UBM is responsible for conducting more current away from the current crowding region, and when the current flows to the other side of the UBM, it returns into the solder and causes secondary current crowding. Thus both current crowding and secondary current crowding play important roles in our observations. As one can clearly see the remaining contact areas in Cu-SAC-L (Fig. 3(b)) and Cu-SAC-H (Fig. 3(c)) are located in the center-left of the bump interface. It is seen that all samples exhibit the new EM failure mechanism of discrete voids nucleation, growth, and coalescence. However, the following quantitative evaluation of void growth will indicate the very different behavior for different material combinations.

In the microelectronics industry the failure of a solder joint is usually defined by a resistance increase of 20%. In this study, the resistance increase of a single solder bump was not monitored directly because it requires a specific design of circuit. However we are able to deduce the resistance change during the EM testing based on our quantitative measurement of the interfacial void growth induced by EM: the resistance *R* of an object is inversely proportional to its cross-sectional area *A* a resistance increase of 20% corresponds to an area decreases of 16.7%. In other words, the joint is considered to fail when the projected area of voids *A* exceeds 16.7%.

**Figure 4:**
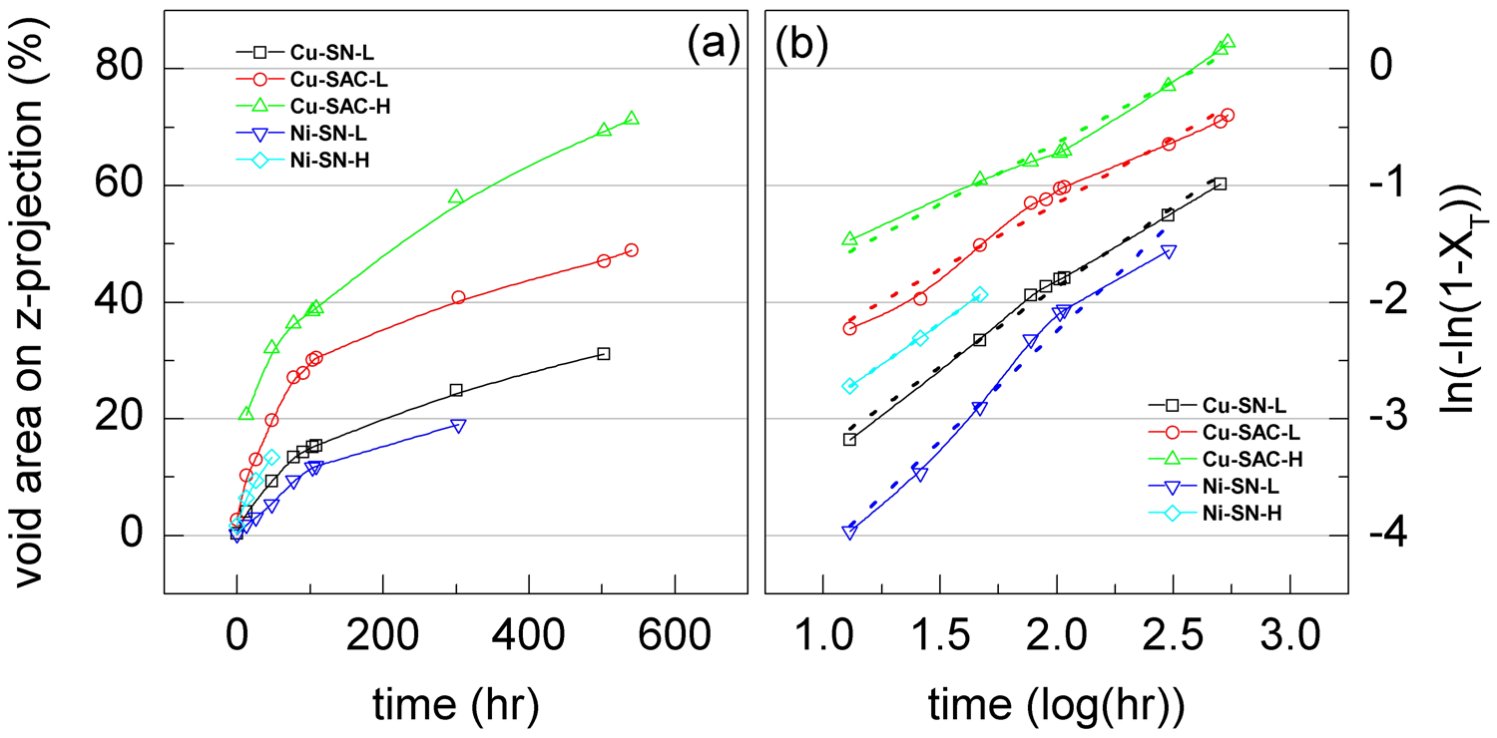
(**a**) The evolution of the projection area of voids onto the UBM opening as a function of the EM testing time for each sample (% of UBM opening area); (**b**) the logarithm plot of the remaining contact area.

The sample of Ni-SN-H reaches open circuit (i.e. failure) after 47.0 hr of EM testing. For the other cases the projected void area in Cu-SN-L, Cu-SAC-L, Cu-SAC-H, and Ni-SN-L reaches 16.7% of the UBM area after 131.5, 44.6, 59.7, and 235.5 hr, respectively, see Fig. 4(a). After these sample failure times EM testing was continued in order to observe the process of microstructure evolution. According to the failure times of all samples, the samples using SAC1205 solder had shorter failure lifetimes. The samples using SN100C survived longer but they had the chance of early failure under a high current stressing density (Ni-SN-H). The difference in the failure times roughly agrees with the reliabilities of the different material combinations^11^. The combination of Ti/Cu/Cu UBM and SAC1205 solder failed first; the combination of Ti/Cu/Cu UBM and SN100C was the intermediate one. Therefore, we can conclude from this study that the effective diffusivity determines the failure time. Since the electroless Ni/Au UBM further suppressed void growth, the combination of electroless Ni/Au UBM and SN100C solder survives without failure for the longest time.

The void nucleation and growth can be viewed as a kind of phase transformation following the JMA theory^11^.

6 $${X}_{T}=1-\exp (-{X}_{{\rm{ext}}})$$

where *X*_*T*_ is fraction of the volume transformed and *X*_ext_ is the fraction of the extended volume, defined as

7 $${X}_{{\rm{ext}}}={\int }_{\tau =0}^{\tau =t}\frac{4\pi }{3}{R}_{N}{R}_{G}^{3}{(t-\tau )}^{3}d\tau $$

where *R*_*N*_ is the nucleation rate, *R*_*G*_ is the growth rate of spherical transformed particles (which refer to voids in this study), and *t* is the time of transformation. Since *X*_*T*_ is the ratio of contact area transformed into voids at testing time *t*, then 1-*X*_*T*_ represents the remaining contact area. Figure 4(b) shows the relation between the remaining contact area and the testing time on a logarithmic axis. All lines in Fig. 4 (b) are approximately linear which indicates that the void formation follows the JMA theory, describing a homogeneous solid phase transformation. This however is very unusual since the original JMA model assumes a fixed driving force for transformation. Instead, the void growth rates in our case decrease with time as shown in Figs 2(a,b) and 4(a). In the kinetics of the JMA model the transformation follows an S-shaped curve where the transformation rate is low at the beginning and the end of the transformation. The initial slow rate is due to the time required for a significant number of new phase to nucleate; the end is due to the limited remaining area for the new phase to form as well as the coalescence between existing new phases hindering the growth. In our study, the void nucleation rate is proportional to the current density^19^. As long as the global current density distribution stays almost unchanged the nucleation rate is almost constant. Therefore our result follows well the JMA model in the beginning of the EM damage i.e. up to 108 hours. Further, at the end of our EM tests the maximum transformation for Cu-SAC-L and -H, Cu-SN-L, Ni-SN-L and -H were only 50%, 70%, 30%, 20%, and 17%, respectively. Thus it is not the amount of untransformed area restricting the transformation rate, but probably the decreasing void growth rate, which is responsible for the slowing down of the total transformation rate. This may lead to the coincidence of our data with the JMA model in the end part of tests. This is a very interesting observation but requires further research. The decreasing void growth rate is attributed to the massive coalescence between voids and the attenuation of the diffusion flux which will be discussed in the following sections.

### The number of voids

**Figure 5:**
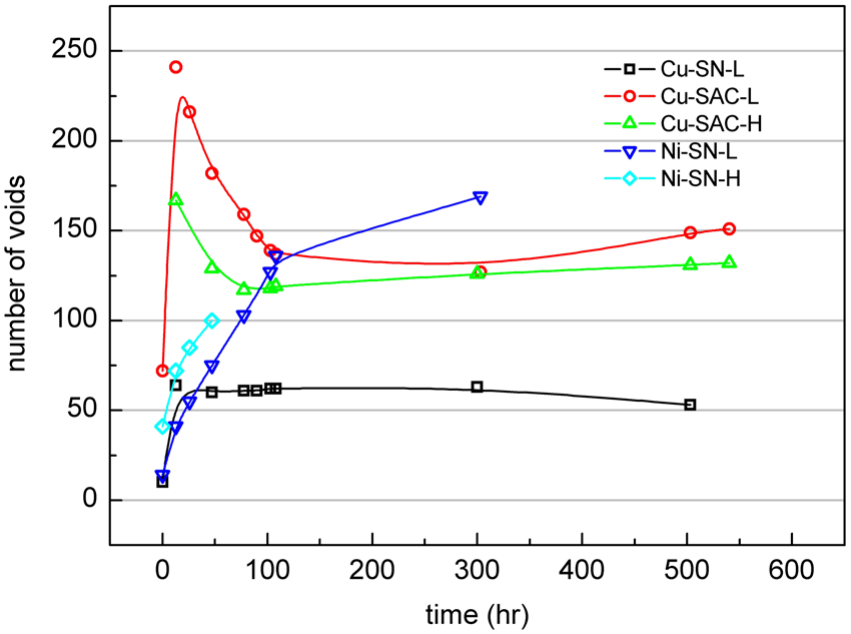
The evolution of the total number of voids during EM testing in all samples.

Figure 5 shows the evolution of the total number of voids during EM testing for all samples. The initial void number in the as-fabricated Cu-SN-L sample before EM testing was only 10, immediately increasing to 64 after the first testing period and then remaining between 60 and 63 for the rest of testing period. In the case of Cu-SAC-L and -H the number of voids also increases significantly at the beginning of tests (72 to 241 in Cu-SAC-L and 167 in Cu-SAC-H), thereafter the void number gradually decreased to 140 (Cu-SAC-L) and 125 (Cu-SAC-H) and remained at these levels. In the case of Ni-SN-L and -H the dramatic increase of the void number in the beginning does not occur, with instead the number of voids continuously gradually increasing during the entire tests.

**Figure 6:**
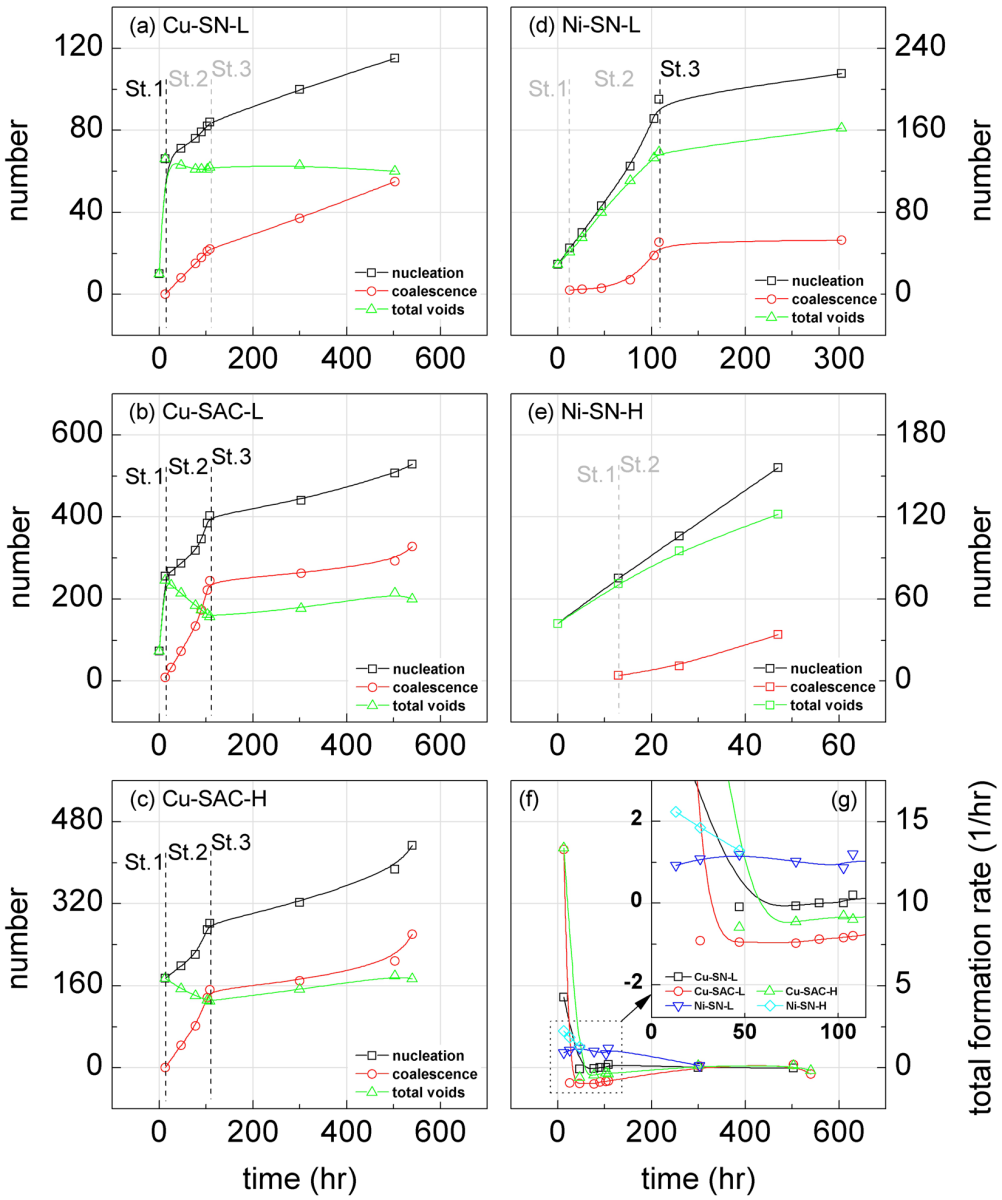
The temporal evolution of the total number of voids (green) along with the cumulative sum of both the newly-formed voids (black) and of the newly coalesced voids (red) for (**a**) Cu-SN-L, (**b**) Cu-SAC-L, (**c**) Cu-SAC-H, (**d**) Ni-SN-L, and (**e**) Ni-SN-H; (**f**) the evolution rate of the total number of voids between adjacent EM stages; (**g**) the enlarged plot of the dashed rectangle in (*f*).

Figure 6(a) to (e) show for each sample the statistics of the total number of voids along with the cumulative sum of the number of newly-formed voids and the cumulative sum of the number of coalescence events between voids. The total number of voids (green line) corresponds to the cumulative numbers of newly-formed voids (black line) minus the cumulative numbers of coalescence (red line). The formation of a new void causes the black line and the green line going up by one in Fig. 6; and a coalescence between voids causes the green line going down by one while the red line going up by one. If all voids grow without any nucleation and coalescence, all three lines stay the same. On one hand the rate of newly-formed void is determined by (1) the void nucleation rate and (2) the magnitude of applied current in this study. On the other hand, the rate of coalescence depends on (1) the growth rate of voids, (2) the spatial density of voids, and (3) the non-uniformity of void distribution. Since the area of UBM in this study is a constant, the average density of voids is proportional to the number of voids. The voids in a crowded region have more chance to coalesce since their mutual distance is reduced. The current crowding effect induced (due to the unique line-to-bump geometry in the flip-chip) can cause such non-uniformly distributed void nucleation. In the following paragraphs the entire testing procedure is divided into three stages: 1st stage from 0 to 13.0 hours, 2nd stage from 13.0 to 108.0 hours, and 3rd stage from 108.0 to 540.3 hours.

As seen from Fig. 6, in the Ti/Cu/Cu 7.5 μm UBM samples (Cu-SN-L, Cu-SAC-L, and -H), the total number of voids increases abruptly in the 1st stage. These voids activate the coalescence between voids from the 2nd stage so the number of coalescing voids begins to increase gradually. In Cu-SN-L the rate of coalescence only balances the rate of newly-formed voids because of the lower DZ* value of the SN100C solder. Thus the number of voids is maintained between 60 and 65. The boundary between the 2nd and 3rd is less clear in Cu-SN-L. In Cu-SAC-L and -H, the solder was SAC1205 whose DZ* value is twice that of SN100C solder: the Sn atom diffuses much faster in SAC1205, causing a higher void growth rate and a higher rate of coalescence of voids. The higher coalescence rate between voids causes the total number of voids to decrease in the 2nd stage in Cu-SAC-L and -H. In the 3rd stage, the number of both newly formed voids and coalescing voids decreases.

Figure 6(f) shows the evolution rate of the total number of voids, i.e. the rate of cumulative newly-formed voids minus the rate of cumulative coalescence. In Cu-SN-L the new void formation rate and coalescence rate are balanced in the 2nd and 3rd stages. The Cu-SN-L curve is high at the beginning of testing (1st stage) and drops to almost zero. Most of the void growth comes from the growth of existing voids. In Cu-SAC-L and -H, the coalescence rate was constantly higher than the new void formation rate in the 2nd stage due to the high formation rate of new voids in the 1st stage. In contrast, the rate of newly-formed voids was always larger than the coalescence rate in Ni-SN-L and -H. Thus the total number of void increases linearly while, because of the slower diffusion rate of Sn in SN100C, individual voids do not grow much in size. By comparing the above observations, a couple of short conclusions can be drawn. Firstly, the electroless Ni/Au UBM suppresses the individual void growth, which, in contrast, is favored by the Ti/Cu/Cu UBM. Secondly, SAC1205 further promotes the void growth rate and consequently raises the coalescence rate, which in turn decreases the total number of voids in the middle stage of testing. In contrast, SN100C does not significantly accelerate the void growth, so that the number of voids reaches a balance in the middle stage of testing.

In the Ni-SN-L and -H samples the solder employed was SN100C, as was used in Cu-SN-L. The same solder implies the same diffusivity of Sn so the difference in void growth behavior stems from the different UBM. In the case of Ni-SN-L and -H electroless Ni/Au 5.0 μm was used. The resistivity of Ni and the corresponding IMC (Ni3Sn4), 6.8 Ω-cm and 28.5 Ω-cm, are much higher than the resistivity of Cu and its corresponding IMC (Cu6Sn5), 1.7 Ω-cm and 17.5 Ω-cm, respectively^44^. The high resistivity UBM and IMC between the Al pad and solder can relieve the current crowding^45^. For this reason the initial abrupt increase of void number is not observed in Ni-SN-L and -H. Therefore, in Fig. 6(d) and (e), the boundary between the 1st and 2nd stage is less clear.

**Figure 7:**
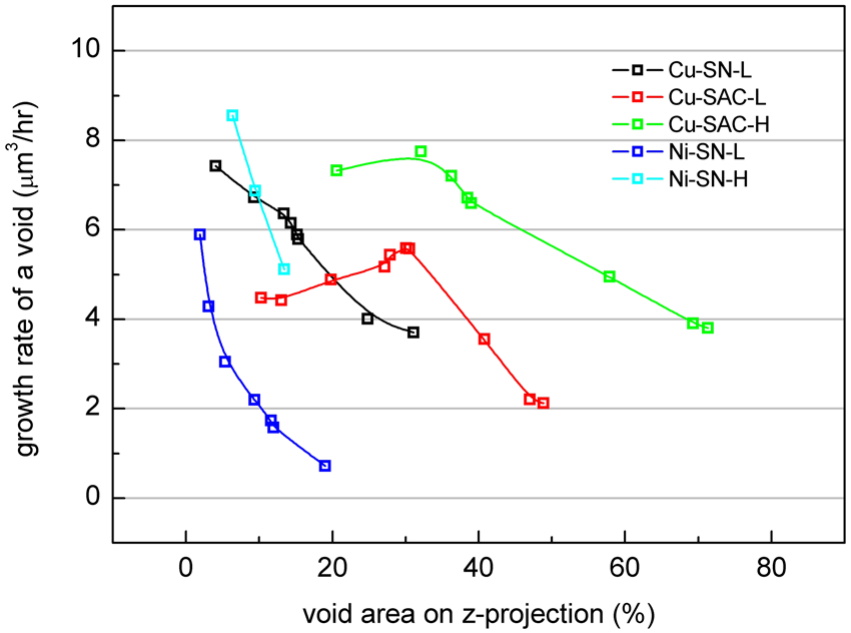
The average volume growth rate of every individual void.

The average volume growth rate of every individual void (shown in Fig. 7), defined by the growth rate of total void volume divided by the total number of voids at each EM stage, indicates the same phenomenon. Since the total void growth follows the JMA theory the growth rate of all voids gradually decreases in all samples. In the case of Cu-SN-L, Ni-SN-L and Ni-Sn-H the total number of voids continued to increase throughout the entire tests, while in Cu-SAC-L and -H it decreases in the 2nd stage of tests. In the 2nd stage of tests, the large voids came from both of the growth of each individual void and the coalescence between them. Therefore, an increase in the growth rate of individual void in Cu-SAC-L and -H in the 2nd stage of tests were observed. Finally, when most of the global current crowding region is occupied by voids the void growth and coalescence slow down and a decrease in the growth rate of individual void in Cu-SAC-L and -H is also seen.

### The correlation between current density distribution and void formation

**Figure 8:**
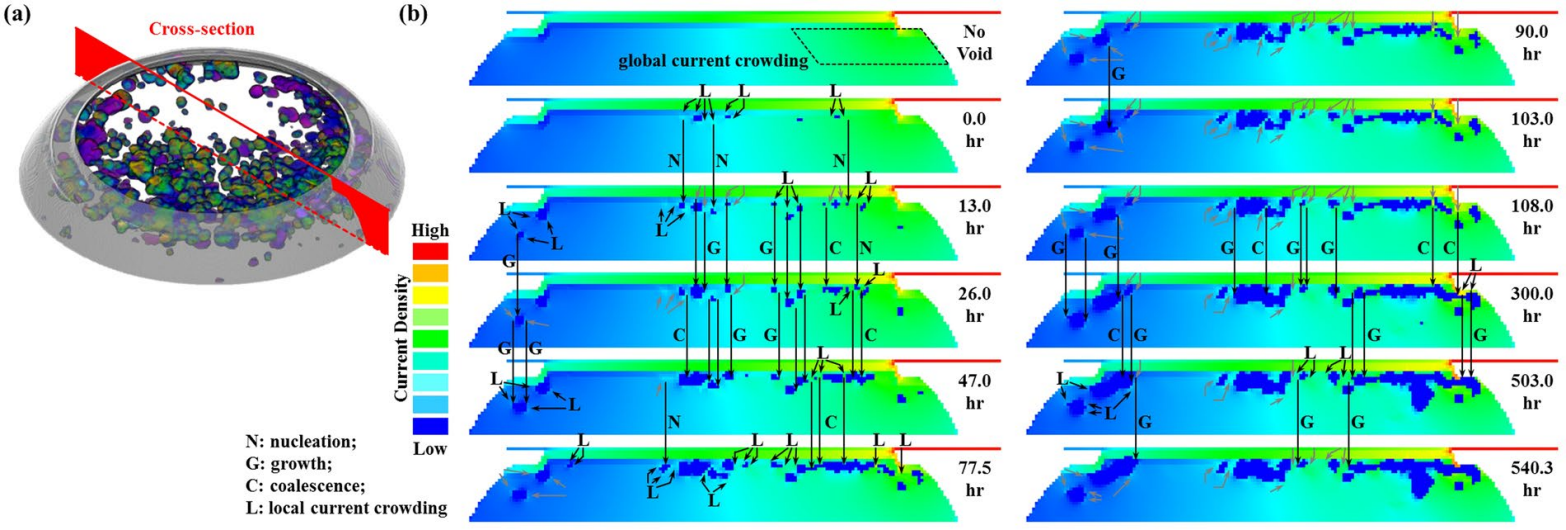
(**a**) The diagonal 3D view of discrete voids in Cu-SAC-L; (**b**) the microstructure evolution and the corresponding current density distribution at the cross-section indicated in (*a*).

Figure 8 shows the evolution of the current density distribution with the void growth in Cu-SAC-L sample during the EM testing. As seen from the color gradient from green to blue in the solder, the global current density distribution across the entire contact area does not change significantly during the EM testing. This gives support to the finding in previous section that the growth of projected void area follows the JMA theory. Apart from the unchanged global one, the effect of local current crowding induced by the discrete voids is observed. This is because current is diverted around the individual voids causing the surrounding area to become locally high current density regions^46,47^. The void formation refers to the accumulation of vacancies, or the diffusion flux divergence. During EM testing the downward electron flow drives the atoms of UBM and IMC into the IMC/solder interface where atomic flux divergence occurs^48–50^. When the supply of atoms at the interface is less than the consumption, voids form. Once a void is formed the consequent local current crowding around the void promotes the growth along the IMC/solder interface. Therefore regions of local current crowding show higher potential for void nucleation, growth, and coalescence. Most of the voids observed during the EM testing are distributed horizontally along the interface.

**Figure 9:**
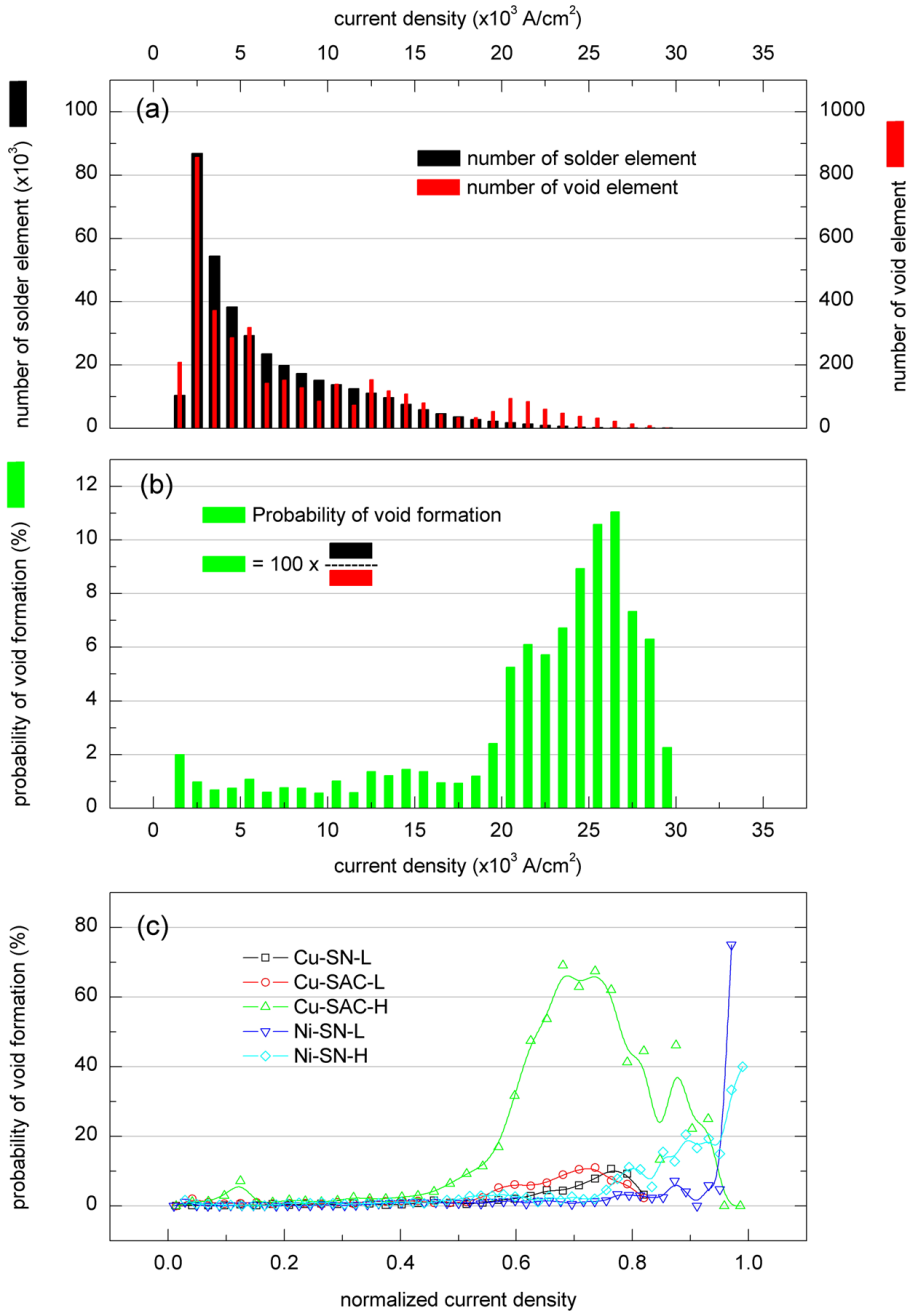
(**a**) The number of solder elements at 0 hr and the number of void elements at 108.0 hr according to the global current density distribution in the Cu-SAC-L sample, (**b**) the probability of void formation with respect to the global current density distribution in Cu-SAC-L sample after EM testing for 108.0 hr; (**c**) the probability of void formation at 108.0 hr for all types of samples with normalized current density distribution.

In order to analyze the effect of the global current density distribution on void formation, Fig. 9 was obtained. The black columns in Fig. 9(a) represent the number of solder elements in the Cu-SAC-L sample before EM testing, the red columns represent the number of void elements transformed from solder elements after EM testing for 108.0 hr. The probability of void formation can be expressed by

8 $${P}_{{\rm{v}}{\rm{o}}{\rm{i}}{\rm{d}}{\rm{f}}{\rm{o}}{\rm{r}}{\rm{m}}{\rm{a}}{\rm{t}}{\rm{i}}{\rm{o}}{\rm{n}}}({\rm{ \% }})=100\times \frac{{E}_{{\rm{v}}{\rm{o}}{\rm{i}}{\rm{d}}{\rm{a}}{\rm{t}}{\rm{c}}{\rm{u}}{\rm{r}}{\rm{r}}{\rm{e}}{\rm{n}}{\rm{t}}{\rm{s}}{\rm{t}}{\rm{a}}{\rm{g}}{\rm{e}}}}{{E}_{{\rm{s}}{\rm{o}}{\rm{l}}{\rm{d}}{\rm{e}}{\rm{r}}{\rm{a}}{\rm{t}}{\rm{p}}{\rm{r}}{\rm{e}}{\rm{v}}{\rm{i}}{\rm{o}}{\rm{u}}{\rm{s}}{\rm{s}}{\rm{t}}{\rm{a}}{\rm{g}}{\rm{e}}}}$$

where *E*_void at current stage_ is the number of void elements transformed from solder elements at the current stage, and *E*_solder at previous stage_ is the number of solder elements at the previous stage. The probability of void formation in the Cu-SAC-L sample and in all samples after EM testing for 108.0 hr is shown in Fig. 9(b) and (c) respectively. These figures indicate that the probability of void formation in all samples is only enhanced when the current density exceeds a threshold value. In Cu-SAC-L the threshold value was roughly 2.0 × 10^4^ A/cm^2^. However, it has to be noted that the threshold value here is not the critical current density in EM^51–53^. The critical current density is an important value in studying EM. It can be obtained by balancing the EM and the resulting back stress. Once a current is applied to a subject, the atoms diffuse from the cathode to the anode. The accumulation of atoms at the anode induces a back stress gradient and the diffusion caused by the stress gradient retards the EM diffusion. The EM damage does not occur in a sample with specific length where the current density is lower than the critical current density. The critical current density is around 1.0 × 10^4^ A/cm^2^ for a 300 μm Sn stripe and a SnAg3.8Cu0.7 stripe^52,53^. The phenomenon shown in Fig. 9(b) and (c) may arise from the combined effect of critical current density and the interfacial reaction between IMC and solder, confirmation of which however requires further studies.

**Figure 10:**
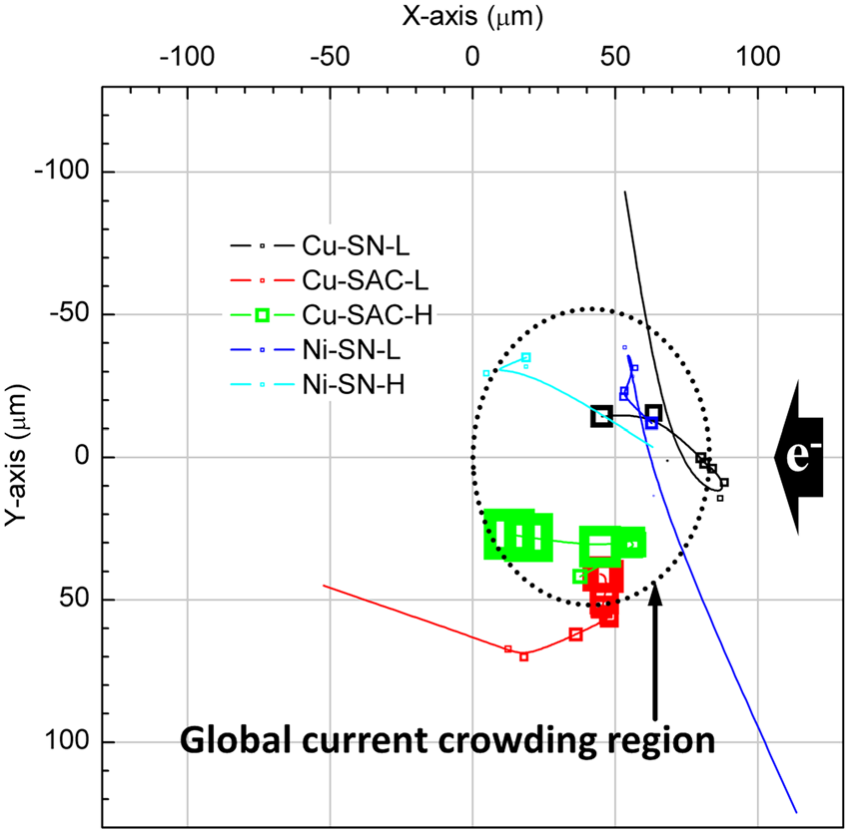
Movement of the geometric center (center of mass) of all voids. The size of dots indicate the size of projected area of the voids on the UBM opening for each EM testing time. The XYZ coordinates are given in Methods.

The movement of the void geometric center (Fig. 10) also indicates the correlation between the microstructure evolution and the distribution of global current density. In Fig. 10, the curves represent the movement of the void geometric center with the dot size indicating the relative value of the total volume of voids at a specific EM testing time. At the global current crowding region the void formation is promoted by the high current density, resulting in the geometric center moving in the positive X-direction in the early stages of testing. After a certain period, discrete voids grow and coalesce into large voids which cover most of the global current crowding region. Subsequent void growth toward the left-hand side of the bump, results in the void geometric center moving in the negative X-direction. This process can be observed significantly in the Cu UBM samples but not the Ni UBM samples because their microstructure evolution was mainly dominated by void nucleation without coalescence after the 2nd stage (as discussed in Fig. 6).

### Vertical void growth caused by thermomigration

**Figure 11:**
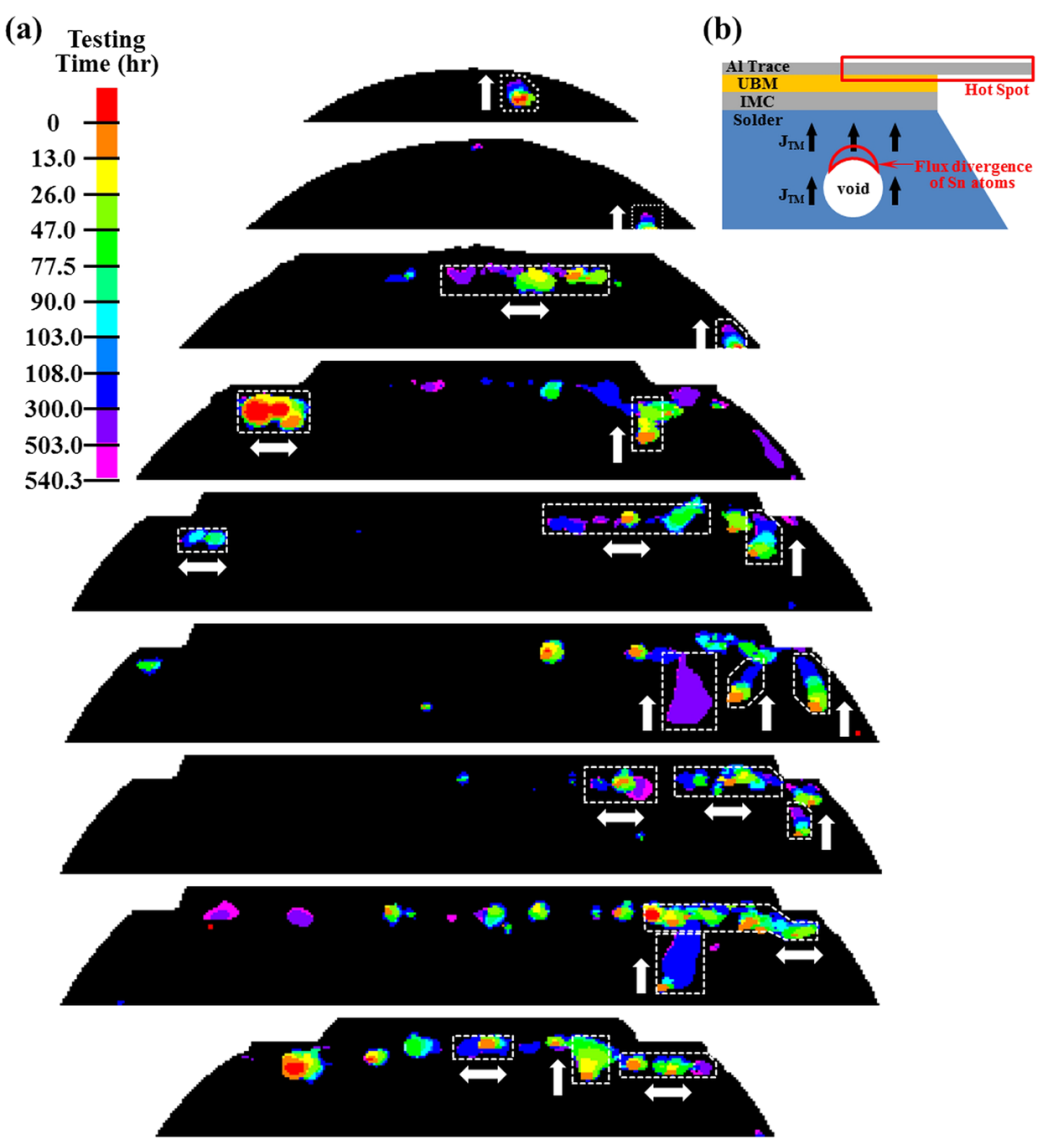
(**a**) The vertical growth of some voids induced by thermomigration in Cu-SAC-L. The top portion of the bump is shown in different vertical cross-sections, the dashed rectangles and corresponding arrows highlight several typical void growth in vertical or horizontal directions; (**b**) the model illustrating the vertical void growth.

A number of voids were observed to grow upward in Cu-SAC-L and -H samples as shown in Fig. 11. From the viewpoint of EM, the voids should grow horizontally along the IMC/solder interface because of the microstructure and the impact of local current crowding. Therefore, these upward growing voids were most probably caused by thermomigration (TM) according to the direction of this driving force^54^. As Fig. 11(b) shows, when the top trace is conducting high current, a large amount of Joule heating is generated and the region near the top trace becomes a hot site. Due to the thermal gradient, a TM flux of Sn is generated toward the hot side since the Q* value of Sn atoms is negative^55–57^. (Heat of transport, Q*, is the difference between the heat carried by the moving atom and the heat of the atom in the initial state). As a result a void can grow upwards towards the hot side of the bump. Since this observation was only found in Cu-SAC-L and -H samples it implies that SAC1205 solder is more sensitive to TM compared with SN100C solder. However the influence of TM on the EM results is assessed to be small since only a few vertically growing voids were observed.

## Discussion

The established pancake void model of flip-chip solder joint failure postulates that only one void forms at the current crowding spot and propagates without coalescence along the IMC/solder interface^13–16,41^. This occurs when the UBM is very thin and the contact resistance is low, and results in a single nucleation and growth event. The basic concept of this model is partially correct when it is applied to the present study, where multiple nucleation events occur. This is possibly because a much thicker UBM is used, so that current crowding is spread across the entire contact area. It seems that the bump design may play a role in determining failure mechanisms, however this requires additional investigations, for example the investigation of similar solder alloys with different mechanical structure using the same observation technique. Nevertheless, this new EM failure mechanism (discrete void growth and coalescence) observed by 3D laminography implies several important differences from the pancake void model. Firstly, the current crowding effect does not limit the void number into only one, although void has higher probability to nucleate in the global current crowding region. However, the correlation only occurs in the high current density region as shown in Fig. 9. Due to the current crowding effect a high current density can be observed in a few samples while in most regions, the current density was lower than the average value, 7.5 × 10^3^ A/cm^2^. All the curves in Fig. 9(c) show the same tendency: the probability of void formation increases considerably when the current density exceeds a certain value. These values do not correspond to the critical current in EM because there will be no EM flux once the applied current is smaller than the critical current^42,51,58^. This is a very interesting phenomenon but requires further studies.

Secondly, the formation of a large void does not only depend on the growth of a small void but also depend on the coalescence of small voids. So the old model cannot reliably estimate the failure rate observed here. In addition, voids nucleate across the entire contact area as shown in Fig. 9(b), not only at one edge of the contact. Some of them are located outside of the current crowding region.

Thirdly, the old model presumes that the pancake void blocks the shortest route for current, diverts it to the growth front of the pancake void, which possesses a wider area, therefore relieving the current crowding effect itself. As a result, the relief of the current crowding effect slows down the growth rate of the pancake void^59,60^. However, the results of the FE models based on the 3D laminography images indicate that discrete void formation only induced a local current crowding effect around the voids. The global current density distribution across the entire contact area does not significantly change during the entire EM testing. The decrease of the void growth rate results from the follow-up behavior of IMC growth. The voids only divert the global current significantly after major coalescence takes place amongst the voids in the late stage of EM testing.

## Conclusion

In this study, solder bumps with two kinds of composition and two kinds of UBM were EM tested under two values of current density. To minimize the drawbacks of the destructive observation method in previous studies, the 3D synchrotron laminography imaging was applied. A new EM failure mechanism involving discrete void nucleation and coalescence was observed, rather than the two well-known ones: pancake void propagation and UBM dissolution. Instead of the nucleation and propagation of only one void, a large amount of discrete voids nucleate and grow simultaneously. After a period of time these discrete voids coalesce into several large voids, causing the bump to fail. The coalescence rate was affected by the diffusion of Sn atoms. Both the kinetic analysis and the values of DZ* indicate that the diffusion of Sn is much faster in the SAC1205 solder than in the SN100C solder. The dopant in SN100C somehow reduces the diffusion of Sn and retards the growth of voids. Therefore, the increase of total void volume in Ni-SN-L and -H results mainly from the formation of new voids. From the view point of UBM a thicker UBM with higher resistivity relieves the current crowding effect. The higher current crowding caused by the Cu UBM led to a peripheral distribution of the discrete voids. As a result, the combination of Ni UBM and SN100C solder was found to provide the best EM reliability. In addition, discrete void growth and coalescence follow the initial regime of the JMA theory. The number of voids is also significantly affected by the UBMs and solders. Due to the severe current crowding caused by the Cu UBM the number of voids dramatically increases in Cu-SN-L, Cu-SAC-L, and -H samples in the initial 13 hours of tests. After 13 hours, the fast diffusion of Sn atoms in SAC1205 solder induce a large amount of coalescence of discrete voids. Therefore, the number of voids drops from over 200 to 140 in Cu-SAC-L and 125 in Cu-SAC-H. With the correlation between void formation and current density distribution, a threshold value of current density for the void formation was found to be 2.0 × 10^4^ A/cm^2^. This discrete void model was consistent with the pancake void model in the final stage of testing, but provides a more accurate observation of the beginning of testing.

## Methods

We combine laminography, quantitative analysis, and 3D finite-element modeling^19^ to investigate the EM failure process for different solder/UBM combinations. The *in-situ* laminography observation provides 3D images non-destructively, without special preparation (like surface cutting or polishing) of the specimens, thus maintaining realistic boundary conditions during testing. Consequently, the quantitative analysis was performed via proper image segmentation and processing. Furthermore, the complete progress of void formation and merging was followed. Based on the 3D laminography images, several series of FE models were built. Then the current density distribution in all stages of samples was obtained. The interactions between the void evolution and the corresponding current density distribution were further discussed.

Several material combinations were chosen for EM testing. As shown in Table 2, there were two kinds of UBM. The Ti/Cu/Cu UBM was applied in Cu-SN-L, Cu-SAC-L, and -H; and the electroless Ni/Au UBM was applied in Ni-SN-L and -H. The Ti/Cu/Cu UBM was made firstly by sputtering 100 nm of Ti and 300 nm of Cu and followed by electroplating 7.5 μm of Cu. The electro-less Ni/Au UBM was made by electro-less plating of 5.0 μm Ni and 50 nm Au finish. Two kinds of solder were chosen: SN100C solder was applied in Cu-SN-L, Ni-SN-L, and -H, and SAC1205 solder was applied in Cu-SAC-L and -H. The composition of SN100C and SAC1205 solder were respectively Sn, Cu 0.65%, Ni 0.05%, Ge 60 ppm, Bi 110 ppm, Pb 140 ppm and Sn, Ag 1.2%, Cu 0.5%, Ni 0.05%.

**Table 2:** The configuration and experimental condition of all samples.

Sample	UBM (μm)	Solder	Current Density (A/cm^2^)
Cu-SN-L	Ti/Cu/Cu 7.5^*a*^	SN100C^*c*^	7.5 × 10^3^
Cu-SAC-L	Ti/Cu/Cu 7.5^*a*^	SAC1205^*d*^	7.5 × 10^3^
Cu-SAC-H	Ti/Cu/Cu 7.5^*a*^	SAC1205^*d*^	1.0 × 10^4^
Ni-SN-L	E-less Ni/Au 5.0^*b*^	SN100C^*c*^	7.5 × 10^3^
Ni-SN-H	E-less Ni/Au 5.0^*b*^	SN100C^*c*^	1.0 × 10^4^

^a^Ti seed layer 100 nm/Cu seed layer 300 nm/Cu UBM 7.5 μm.

^b^Electro-less Ni UBM 5.0 μm/Au layer 50 nm.

^c^Sn, Cu 0.65%, Ni 0.05%, Ge 60 ppm, Bi 110 ppm, Pb 140 ppm.

^d^Sn, Ag 1.2%, Cu 0.5%, Ni 0.05%.

The samples for the EM tests were produced by National Semiconductor Corporation. There were four identical wafer-level chip-scale-packaging chips mounted on a single printed circuit board. In each chip (3000 μm × 3000 μm) a total of 36 solder bumps were fabricated. By varying the under-bump-metallization (UBM), the solder material, and the magnitude of the applied current, five different sample conditions were tested. The details are listed in Table 2.

By controlling the magnitude of applied current, the current density on the UBM opening in Cu-SN-L, Cu-SAC-L, and Ni-SN-L was 7.5 × 10^3^ A/cm^2^, and in Cu-SAC-H and Ni-SN-H it was 1.0 × 10^4^ A/cm^2^. During EM testing, a furnace and a thermal couple were used to set and monitor the surface temperature of the chip at 130 °C.

**Figure 12:**
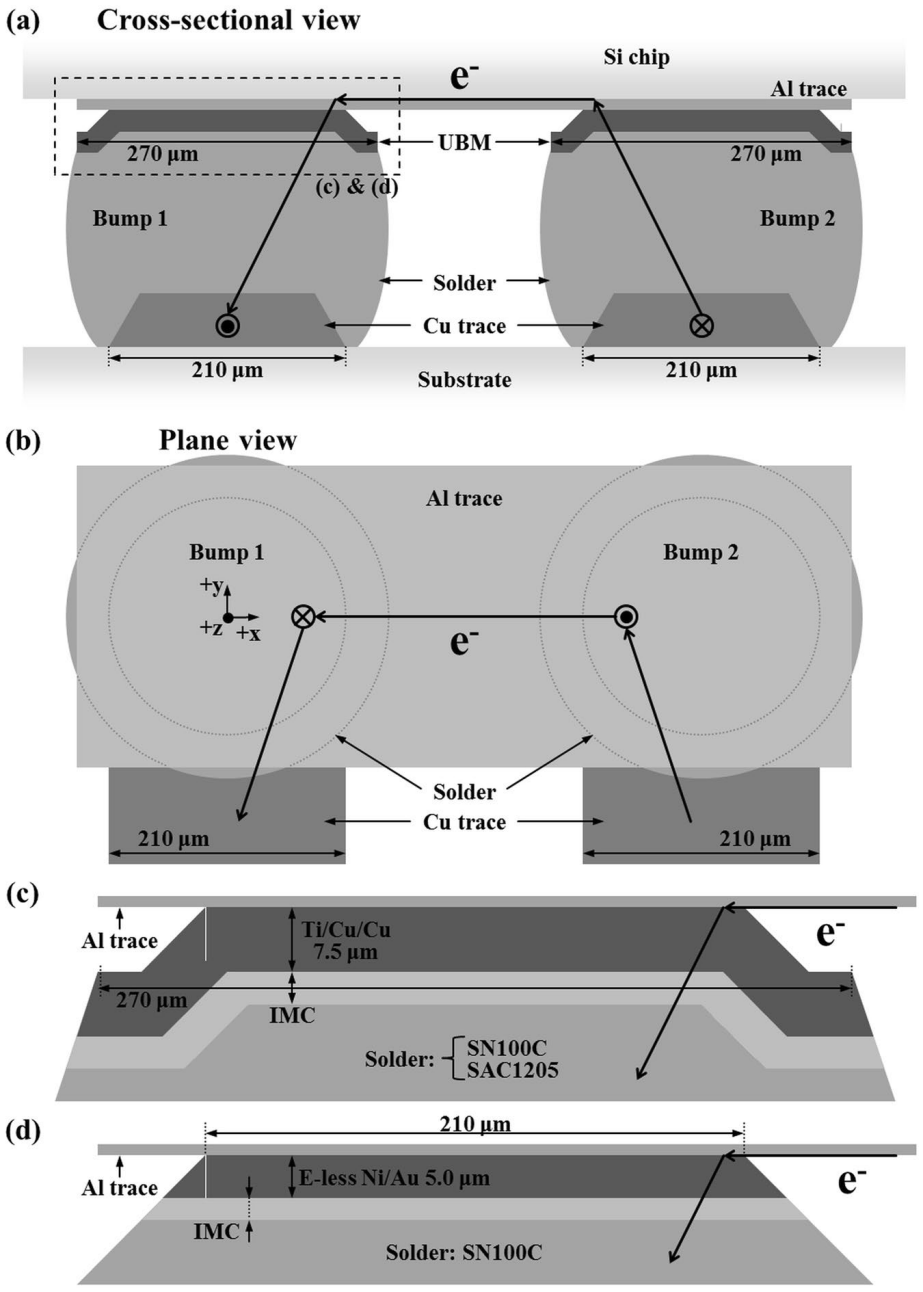
(**a**) The schematic cross-sectional view (XZ) and (**b**) the plane view (XY) of the sample structure; the enlarged image of the dashed rectangular region in (*a*) for (**c**) Cu-SN-L, Cu-SAC-L and -H samples and for (**d**) Ni-SN-L and -H samples, which are the major regions of interest in our study.

The sample structures were schematically illustrated in Fig. 12. There was one pair of solder bumps (Bump 1 and Bump 2) tested in each sample, as shown in Fig. 12(a) and (b). Bump 1 which was conducting a downward electron flow underwent more damage because the top trace is much thinner than the bottom one, as a result inducing a higher current crowding effect. The other bump with the opposite electron flow direction is labeled as Bump 2. Particularly, the top UBM/solder interface of Bump 1 where a high flux of electrons entered is the major region of interest (ROI). Figure 12(c) and (d) are the enlarged illustration of the dashed rectangle in Fig. 12(a). The diameters of passivation opening, UBM opening, and bump height are 210 μm, 270 μm, and 220 μm respectively. The top trace was 1 μm thick Al, and the bottom trace was Cu which provide good adhesion.
